# Safer cycling in older age (SiFAr): a protocol of a randomized controlled trial

**DOI:** 10.1186/s12877-021-02502-5

**Published:** 2021-10-12

**Authors:** Hanna Maria Siebentritt, Veronika Keppner, Sabine Britting, Robert Kob, Anja Rappl, Cornel C. Sieber, Ellen Freiberger

**Affiliations:** 1 Institute for Biomedicine of Aging, Friedrich-Alexander-Universität Erlangen-Nürnberg, Kobergerstraße 60, 90408 Nürnberg, Bavaria Germany; 2 Department of Medical Informatics, Biometry and Epidemiology, Friedrich-Alexander Universität Erlangen-Nürnberg, Erlangen, Bavaria Germany; 3 Department of Medicine, Kantonsspital Winterthur, Winterthur, Switzerland

**Keywords:** Aging, E-bike, Cycling safety, Cycle training, Mobility, Randomized controlled trial

## Abstract

**Background:**

Cycling has positive effects on health and the proportion of older cyclists is rising. However, the risk for older adults to be injured or killed by a bicycle accident increases. The aim of the ongoing project “Safer Cycling in Older Age (SiFAr)” is to promote safer cycling in community-dwelling older adults with a structured, multi-component exercise training.

**Methods:**

SiFAr is a randomized, controlled trial with a duration of 3 months for the intervention and a 6–9 months follow-up. We address community-dwelling persons aged 65 years and older living in the area Nürnberg-Fürth-Erlangen (Germany) who are either 1) beginners with the e-bike or 2) feeling self-reported unsteadiness when cycling or 3) uptaking cycling after a longer break. Long-term, experienced cyclists without subjectively reported limitations or worries when cycling are excluded. Participants are either randomized 1:1 to an intervention group (IG; receiving multi-component exercise program related to cycling, MEPC) or an active control group (aCG; receiving health and bicycle-related presentations, HRP). The purpose of this study is to investigate if the cycling competence of the IG will improve compared to the aCG. The cycling competence as primary outcome is tested not blinded in a standardized cycle course prior and after the intervention period, which consists of variant tasks requiring motor and cognitive skills related to traffic situations in daily life. Additional assessments such as physical functioning, quality of life, fear of falling, questionnaires regarding cycling behavior are obtained.

To investigate the primary objective, regression analyses with difference of errors in the cycling course as independent variable and group as dichotomous dependent variable adjusted for covariates (sex, bicycle type) will be performed.

The trial design is described in the present manuscript, using the extended CONSORT checklist for reporting pragmatic trials.

**Discussion:**

Since there is a lack of cycling-related interventions for older people, SiFAr aims to evaluate a standardized intervention to enhance cycling safety. The results of the SiFAr trial could contribute to the implementation of an evaluated cycling course concept promoting mobility and independence of older adults.

**Trial registration:**

This study was registered with clinicaltrials.gov: NCT04362514 on April 27, 2020

**Supplementary Information:**

The online version contains supplementary material available at 10.1186/s12877-021-02502-5.

## Background

Independence and mobility into old age is of great individual and social importance, which is not at least reinforced by demographic change. Mobility is often at risk by decreasing physical and cognitive capacities as well as functional impairments. Regular physical activity is a key factor for counteracting age-related decline of physical and cognitive functions. Cycling is an affordable, environmentally friendly and convenient form of physical activity that is associated with health and functional benefits, even for older adults with chronic conditions [1, 2]. Several studies showed a positive effect of cycling on cardiovascular health, quality of life [3], fear of falling [4], functional and cognitive status and metabolic responses in middle-aged and older persons [2, 5]. In addition, cycling in older age can improve balance [4, 6] and executive functions [7] and reduce the risk of all-cause mortality [8]. A recent systematic review including observatory and experimental studies suggests with moderate evidence that even a regular use of E-Bikes promotes cardiorespiratory fitness [9].

In Europe, the sales figures of bicycles are increasing in the last years due to the growing popularity of electrically assisted bicycles (e-bikes^1^) [10, 11]. In 2019, 1.360.000 e-bikes were sold in Germany, representing a market share of 31.5% of all bicycles [12]. A German mobility study by the Federal Ministry of Transport and Digital Infrastructure report that half of all e-bike routes were used by persons aged 60 years and older [13]. The 10reasons for the use of e-bikes are the ability to cycle with less effort and to bike longer distances for both leisure and commuting purposes, accompanied by health and environmental aspects [14, 15].

Despite the positive trend of growing cycling-popularity and the health benefits mentioned, cycling poses potential risks for older adults [16]. Data from different European countries also indicates that older cyclists have a higher risk for bicycle accidents leading to serious [17] or fatal injuries [18]. A report of the European Commission [18] based on the “Community database on road accidents” (CARE) including data from 2014 showed that 44% of fatal bicycle accidents happen to persons who are 65 years and older. In Germany, the population-based risk for older adults of having an bicycle accident causing injury or death has increased by 80.1% from 1980 to 2019 [19]. About 58.7% of cyclists fatally injured were 65 years and older, which might be associated with a higher e-bike use in this age group compared to younger persons. The proportion of e-bike riders in fatally injured cyclists was 19.9% in 2019, while the proportion of seriously and lightly injured e-bikers was 15.4 and 10.2%, respectively [20]. The higher vulnerability caused by the age-related decline of physical and cognitive function might essentially affect the ability to avoid accidents and safe cycling behavior (e.g. reaction, coordination, motor competence). In order to counteract the increasing number of serious and fatal injuries among older adults, effective training interventions are needed to improve safe cycling skills in older persons.

Existing cycling-related intervention studies with older adults focused on investigating the effect of cycling on health outcomes (e.g. cognitive function, well-being [7, 21]) or on specific functional abilities (e.g. muscle strength and balance [4, 6, 22]), whereas the effect on cycling skills and behavior is mainly determined in studies with children [23–25]. There is a lack of representative intervention studies aiming at improving the specific physical and cognitive skills required for safer cycling behavior among older people. In Germany, there are several concepts for safe cycling behavior, such as the “moveo ergo sum” program of the Association of Cycling Instructors (VdR) or the cycling schools of the General German Cycling Club (ADFC), but they do not focus on the specific needs of older people. Furthermore, these training concepts have hardly been scientifically evaluated for their effectiveness. In 2013, the Technical University of Dresden and the University of Leipzig conducted an intervention study to improve the physical condition of cyclists with special focus to the requirements of safe cycling exercise for older adults [26]. The training sessions took place in gyms or fitness centers and included exercises for motor skills, coordination, reaction and balance, but there was no training on the bicycle. Participation in the progressive training program for 6 months twice a week showed no significant effect on the main outcome, the performance in a cycle course. The authors consider that the trainers did not adequately make the link from exercise in the gym to cycling in everyday life. Therefore, no improvements of the performance in the cycle course as a transfer test could be seen.

### Objectives

Therefore, the primary objective of the “Safer Cycling on Older Age” (SiFAr) project of the Institute for Biomedicine of Aging (IBA, University of Erlangen-Nürnberg, Germany) is to investigate if the provision of a structured and progressive multi-component exercise program related to cycling (MEPC) for older adults improves the cycling competence (e.g. balance, strength, ability to react, cycling skills and techniques). The cycling competence is measured by completing various tasks in a cycle course in the intervention (IG) and the active control group (aCG) before and after a three-month training period. The second objective is to examine if the intervention will lead to long-term effects on cycling competence.

### Hypotheses

We hypothesize that the participation in the IG compared to aCG will lead to a reduction of errors in the cycle course reflecting improvement of cycling competence (primary outcome). Furthermore, we hypothesize that a possible reduction of errors in the cycling course due to participation in the IG will have a long-term effect on the cycling competence over 6–9 months.

## Methods/design

### Study design and randomization

**Fig. 1:**
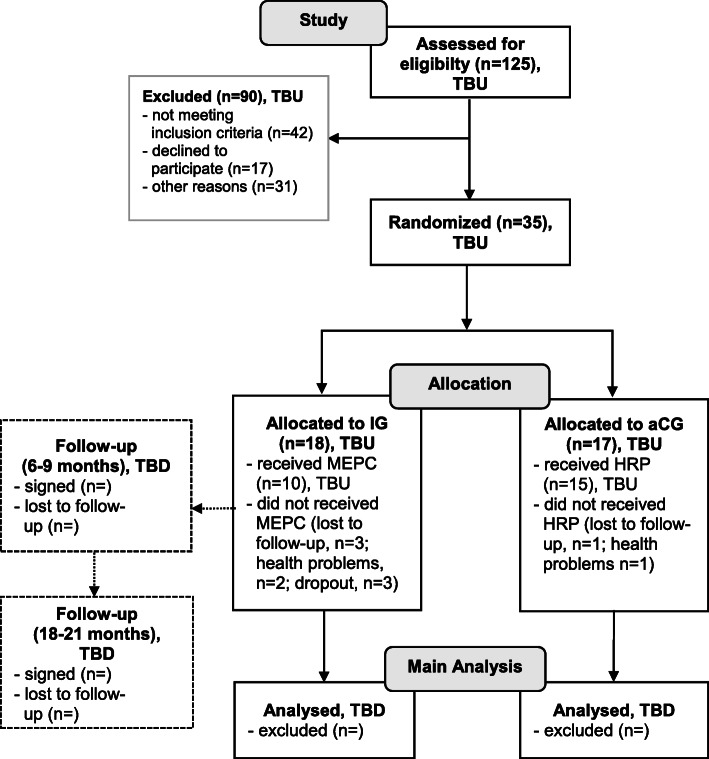
CONSORT 2010 Flow Diagram (Clinical Trial Registration Number: NCT04362514) Status Recruitment 2020.

TBU, to be updated; TBD, to be done; IG Intervention Group; aCG active Control Group; MEPC Multi-Component Exercise Program related to cycling; HRP Health-Related Presentations.

SiFAr is a parallel group, randomized controlled, explanatory, ongoing trial with a duration of 3 years (see Fig. 1). The 1:1 randomization of participants to IG or aCG is stratified by sex and bicycle type (e-bikes/unmotorized bicycle). Furthermore, couples are randomized together to ensure that they could participate in one group. Blocksize for IG and aCG was chosen to be between 2 and 4. The randomization lists were computer-generated via simple random sampling without replacement in the respective strata with the statistical software R 4.0.2^2^ by a statistician who was otherwise not involved in the planning of the study design. Randomization is performed by enrolling participants in concealed randomization lists by trained study personnel after assignment of the informed consent and the baseline assessments during the first in-person visit. All study personnel including examiners is involved in enrolling participants, collecting data, entering data into the database and scheduling participants, and therefore not blinded. All study personnel is carefully trained to ensure the standardization of assessments. However, MEPC is instructed by external cycle trainers who are not involved in enrollment and assessments or any other part of the study procedure. The study design is summarized in the WHO Trial Registration Data Set (see Supplementary file ‘WHO Trial Registration Data Set’).

### Sample size calculation

Sample size calculation was performed using G*Power 3.1.9.2 [27]. The difference of errors in the cycle course between before and after the intervention period will be considered as primary outcome. Since this is the first study on this topic, no reference values were available as to which error difference is to be expected between the study groups. Therefore, we conservatively assumed a mean error difference of 0.7 between the IG and the aCG for this explanatory analysis. The standard deviations in both groups were assumed equal, since we had no reason to expect otherwise. This resulted in an effect size of 0.47. Based on these considerations 200 individuals need to be recruited for the study to detect a reduction of mean error difference of 0.7 attributed to the intervention with 90.7% power at a two-sided level of significance of 5%. We considered different scenarios and calculated power in each. Finally we decided on a very conservative setting, in which we would see sufficient power (over 80%) even if a participant drop-out of the full 25% realized.

### Recruitment and eligibility

The recruitment of the study participants takes place between April 2020 and July 2022 via advertisement in the local media (e.g. radio, newspaper, brochures) and by using a database of the IBA. In addition, bicycle organisations and bicycle dealers are contacted to display flyers or to specifically address potential participants. Due to the corona pandemic, recruitment did not start in April 2020 as originally planned, but in June 2020.

The target population of the study are community-dwelling persons aged 65 and older who are either 1) beginners with the e-bike or 2) feeling self-reported unsteadiness when cycling or 3) uptaking cycling after a longer break. Eligible participants have to live in the area Nürnberg-Fürth-Erlangen, Bavaria, Germany and have to be able to come to the training locations on their own bicycle. Long-term, experienced cyclists without subjectively reported limitations or worries when cycling are excluded. Further exclusion criteria are the presence of diseases (e.g. cardiovascular diseases, severe functional impairment, cognitive impairment, non-compensable hearing or vision loss) that contradict safe participation in the intervention and other factors that prevent regular and safe participation (e.g. prolonged holidays during the training period, alcoholism).

By a systematic telephone interview, study eligibility is screened and individuals meeting the inclusion criteria are invited to the study center.

### Ethics

The study protocol was approved by the ethic committee of the Friedrich-Alexander-Universität Erlangen-Nürnberg, Germany (FAU). The study was registered at ClinicalTrials.gov (identifier: NCT04362514) and the study design takes into account the principles set out in the Helsinki declaration. All participants received a written information sheet containing the most relevant study components and have to sign informed consent forms prior to assessments at the beginning of the baseline visit. Changes to the protocol are reported to ClinicalTrials.gov and approved by the ethical committee. During the period of study participation, all participants are provided insurance for the intervention and all assessments.

### Data collection

The flow of data collection is shown in Table 1. Baseline data collection (T0) takes place in-person in the study center (participants characteristics, functional and psychological assessments) and in the cycle course (performance in the cycle course) with their own bikes on two different days within approximately 14 days. Except for cognitive function, same data are collected of all participants after the 3 months intervention period (T1), regardless of their compliance. In addition, participants will be followed up 6–9 months after T1 (T2) related to seasonal time frames (after winter season). Furthermore, participants of the IG 2020 will be measured for a long-term follow-up 18–21 months after baseline in the third year of the study (T3). Data collection will be finished by the end of 2022.

**Table 1:** Schedule of enrolment, interventions, and assessments of the SiFAr trial

	STUDY PERIOD			
	Enrolment Baseline	Post-allocation		
**TIMEPOINT**	***t***_**0**_	***t***_***1***_	***t***_***2***_	***t***_***3***_
**ENROLMENT:**				
**Eligibility screen**	X			
**Informed consent**	X			
**Allocation**	X			
**STUDY ARMS:**				
***Intervention Group***	X	X	X	X
***Active Control Group***	X	X	X	
**ASSESSMENTS:**				
***Sociodemographic Characteristics***	X			
***Weight/Height/BMI***	X	X	X	X
***Health Parameters (Medication, Diseases, Physical Activity)***	X	X	X	X
***Cognitive Function (MoCA, TMT A&B)***	X		X	X
***Performance in the Cycle Course***	X	X	X	X
***Physical Performance (SPPB)***	X	X	X	X
***Quality of Life (EuroQoL-5D + vas)***	X	X	X	X
***Falls/Fear of Falling (FES-I short form)***	X	X	X	X
***Bicycle-related Parameters (falls, cycled distance, self-reported cycling behavior)***	X	X	X	X

*MoCA* Montreal-Cognitive Assessment [28]; *TMT* Trail Making Test [29]; *SPPB* Short Physical Performance Battery [30]; *VAS* Visual Analogue Scale [31]; *FES-I* Falls Efficacy Scale-International Version [32].

Efforts will be made to obtain reasons for dropout. Data safety and management adhere to the national and European data regulation law (EU-DSGVO). All personal identifying data is saved separately in a password protected file never be shared and deleted after the end of the trial. Data entry is double-checked. All data is stored on the university network storage with a regular back-up.

Possible adverse events (especially cycling accidents and falls) during study participation are recorded.

#### Primary outcome

**Fig. 2:**
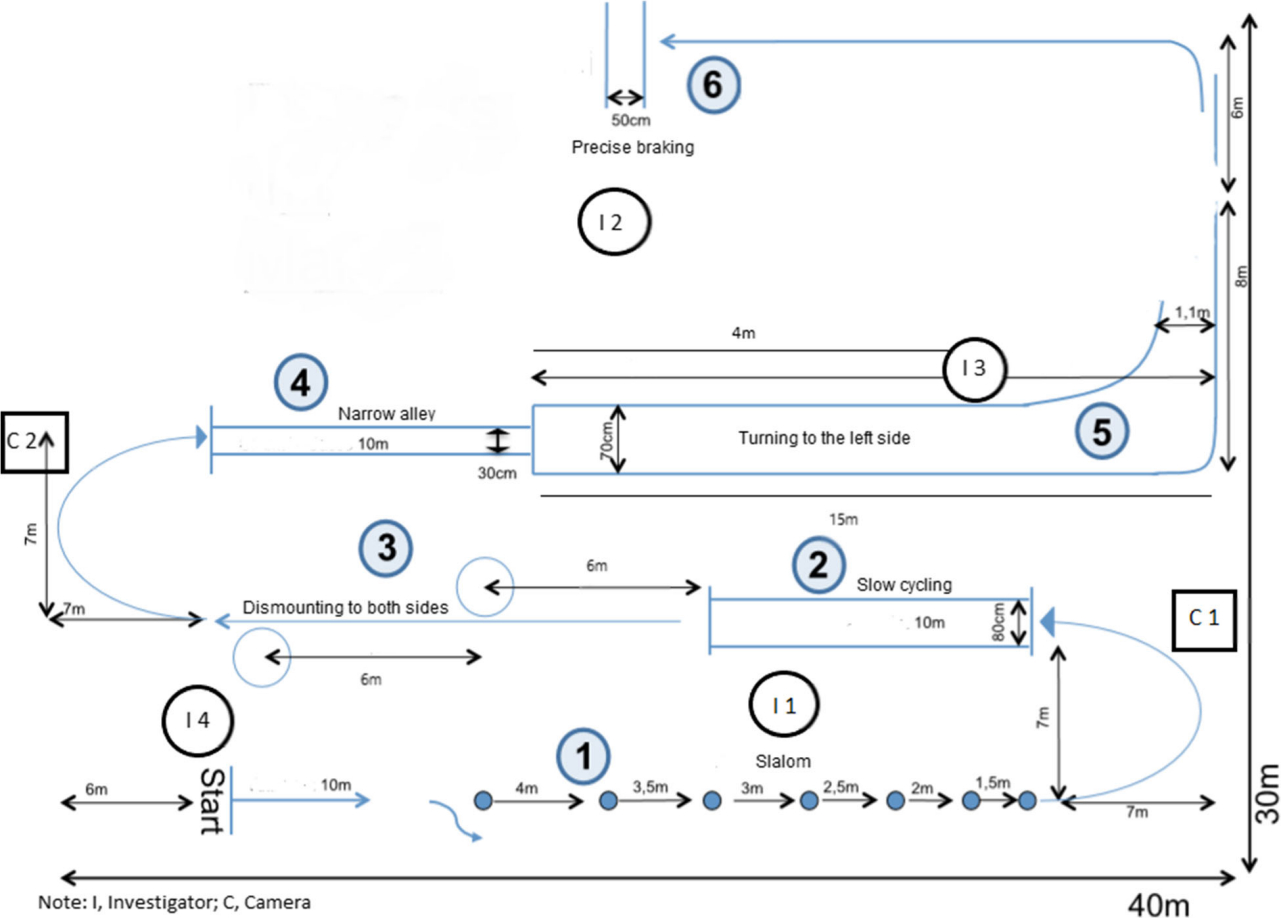
Cycle course (adapted from Hagemeister & Bunte, 2014); I, Investigator; C, Camera

The cycling competence as primary outcome is tested in a standardized cycle course which was developed to test the motor competence of secondary school children after cycling training [33]. If possible due availability, the primary outcome is tested on the same testing location for all participants. Hagemeister et al. [26, 34] showed in their study that the cycle course in an adapted form is also feasible and safe for older adults. In the SiFAr study, a modified version (see Fig. 2) is used including the following 7 tasks: slalom, slow cycling, dismounting into a hula hoop, getting on the bicycle, cycling through a narrow alley, turning to the off-side, precise braking. The respective tasks and possible errors are described in Table 2. Mean change of number of errors in the cycle course will be tested between T0 (baseline) and T1 (after 3 month intervention period).

**Table 2:** Cycle course tasks

Task	Instruction	Rating – yes/no and number of errors
**1. Slalom**	- *entrance from the right side* - *driving through all 7 cones in Slalom-style without touching the cones or the ground*	- wrong direction of entering parcours - touching or missing a cone - pushing with foot or touching the ground - passage in standing position
**2. Slow cycling**	- *passage of the corridor as slowly as possible without touching the marked sidelines or the ground*	- touching a line with the front wheel - touching the ground - passage in standing position - riding too fast (< 5 s)
**3.a Dismounting to both sides into a hula hoop** (right side first)	- *accurate stopping next to the hula hoop* - *both feet must be placed into the hula hoop one after another without touching the ring (leg proximal to the hula hoop must go first)*	- missing or touching the hula hoop - dismounting on wrong side - entering the hula hoop with only one leg before getting on the bicycle - foot not placed directly from the pedal into the hula hoop - tipping over of the bike
**3.b Mounting the bicycle out of the hula hoop from both sides** and initiate to ride	- *leg closer to the bicycle must be moved directly from the hula hoop onto the pedal* - *initiate riding with simultaneously placement of second leg on pedal*	- failing to mount bicycle from a standing position - foot not placed directly from the hula hoop onto the pedal
**4. Narrow alley**	- *passage of the corridor without touching the marked lines*	- touching the ground - touching the line with the front wheel
**5. Turning to the left side**	- *riding straight inside the marked corridor* - *initiation of the turning process by well-timed hand-sign and look over the shoulder* - *capture of the shown number by looking behind* - *turning left within the curved corridor*	- missed look over the shoulder - missed arm signal/hand-sign - missed or erroneous naming of the number - touching the lines with the front-wheel - touching the ground
**6. Precise braking**	- *cycling straight at increased speed* - *precise braking within the corridor (ground-front wheel contact point within the corridor)*	- stopping before or behind the corridor - back wheel fish-tailing - jumping off the bicycle or braking with the feet - slow speed before stopping

Before the actual measurement of cycling performance, the participant walks through the cycle course together with study personnel. The individual tasks are instructed and the participant has the opportunity to ask questions. After a test run on their own bicycle, errors are recorded in the second run by using standardized protocol sheets. At least 5 persons from the trained study staff are present at each test session for detailed detection and monitoring of errors. In addition, the sessions are recorded by 2 cameras to check the protocol sheets against the video material.

### Intervention

#### Intervention group - multi-component exercise program related to cycling (MEPC)

The investigated intervention is a multi-component exercise program with and without bicycles, which is specially tailored to the needs of older persons. Training period lasts over 3 months with 8 sessions à 60 min. The sessions take place once a week on a fixed day and time outdoors. The three-month intervention period provides a buffer to catch up on the sessions in case of cancellations due to bad weather conditions (heavy rain, storm, thunderstorm). The participants use their own bicycles and are considered as “adherent” to the intervention if they have participated in at least 6 of the sessions.

The MEPC focuses on the improvement of motor competence (balance, strength, cycling skills and techniques) and cognitive skills required during cycling. Furthermore, fall-related psychological concerns are addressed. Each session has a thematic focus (e.g. braking, dismounting) and follows the same structure: welcome and brief evaluation of the state of health, balance and strength exercises without bicycle, repetition and consolidation of the contents of the last session with bicycle, teaching of techniques by instructors, practice of the techniques by participants (see Table 3). The sessions aim to train the basic cycling skills required to perform the cycle course, but not one or several tasks of the cycle course itself. During the sessions, a transfer to everyday situations is established and bicycle-related traffic rules are discussed. The participants are given a summary of the most important information in a leaflet and they receive instructions for home exercises, which are discussed in the following session.

**Table 3:** Exercise program

**Session 1:** Mounting/Dismounting the bicycle	
- short introduction of the cycling course - bike check (road safety and ergonomics) - introduction of balance and strength exercises (Semi-Tandem; wide leg squats, 2 × 10 repetitions) - information on general behavior and safety instructions in the cycling course - exercises for getting on and off the bicycle on both sides	
**Session 2:** Braking	
- balance exercises (variations of Semi-Tandem-stand, Tandem-stand and one leg-stand with 2 × 10 s holding time per side) and strength exercises (wide leg squats, hip flection, 2 × 10 repetitions^a^) - consolidation of previous session: getting on and off the bicycle at marked position or on command - exercises for precise braking from different speeds - teaching of braking techniques and environmental influences on braking distance	
**Session 3:** Riding curves	
- balance and strength exercises - consolidation of previous session: braking/getting of the bicycle to various commands, discussing the effects of divided attention - teaching and practicing of curve techniques (different speeds and changing curve radius, avoiding obstacles)	
**Session 4:** Track-keeping	
- balance and strength exercises - consolidation of previous session: riding curves one-handed, ride along a partner - teaching and practicing of techniques to stay on the track: riding through a narrow corridor at various speeds, transfer to real-life situations	
**Session 5:** Turning to the left	
- balance and strength exercises - consolidation of previous session: skill exercises for track-keeping (partner exercises, „snale race“) - teaching and practicing of techniques for turning to the left (hand-sign, looking over the shoulder) while riding in a straight line	
**Session 6:** Turning to the left	
- balance and strength exercises - consolidation of previous session: riding one-handed (hand-sign) and look over each shoulder while riding in a straight line - practicing techniques for the 8 steps of turning left in line with traffic regulations	
**Session 7:** Divided attention/complex situations	
- balance and strength exercises - practicing cycling with additional cognitive tasks (calculation, conversation with partner and memorizing the contents of the conversation) and in complex situations (cycling an 8) - teaching of cycling-related traffic regulations	
**Session 8:** Skills exercises/Practicing	
- balance and strength exercises - teaching of safe traffic behavior (e.g. blind spot, anticipation of mistakes by other drivers) - Skill exercises for integration of practiced driving techniques (e.g. “shadow riding” with partner) - course reflection: exchange of experiences	

^a^since each contains a variation of these balance and strength exercises, these are no longer described in detail for sessions 3–8

The intervention takes place outdoors on different large traffic-calmed community places. This allows the use of aids (cones, hula hoops, lines) and surroundings (narrow paths, small hills). The instructors of the training have either expertise in the field of bicycle courses [trainers of the *German Initiative Mountain Bike* (DIMB) or the *German Alpine Club* (DAV)] or in the field of exercise programs for older persons. Before the start of the intervention, the instructors take part in a two-day training session on the standardized multi-component exercise program. During the intervention period, they are in close contact with the study coordinators, for example to discuss the training condition of the participants. In addition, supervision sessions with all instructors are held at least once during the intervention period for feedback and questions. Adherence to intervention is administered by monitoring weekly attendance. Efforts will be made to identify those individuals who need support and encouragement by ensuring a good participants - study staff relationship. In addition, participants will be contacted when they miss a session without information of a planned absence due to other appointments.

#### Active control group - health related presentations (HRP)

The aCG receives 3 health-related presentations (one per month) with a duration of 60 min at the IBA. The presentations include the topics physiological changes with age, safety check of bicycle and traffic regulations. Due to the corona pandemic in 2020, no lectures can be offered in person. Instead, leaflets with the respective information are sent to the participants of the aCG. In order to control for possible effects of the presentations on the cycling performance, the same topics are part of the MEPC.

After finishing all parts of the aCG (presentations, T0, T1, T2 measurements), participants will be offered the opportunity to attend the MEPC. This will also help the decrease the dropout rate of this group.

### Statistical analysis

Participants’ characteristics will be presented as mean ± standard deviation or median for continuous variables. Dichotomous and categorical variables will be shown as absolute numbers and percentages. Depending on whether normal distribution is present, independent t -test or chi-square test will be used to compare groups at baseline. In order to test the distribution, histograms and box plots will be applied. To investigate the primary outcome, regression analyses of complete cases with difference of errors in the cycle course (absolute difference T1-T0 or relative difference (T1-T0)/T0) as independent variable and group (IG/aCG) as dichotomous dependent variable adjusted for randomization factors (sex, bicycle type) and covariates (e.g. cycled distance within 3 months) will be performed. Prior to the analyses, data will be checked for normality (Q-Q-Plots, histograms) and outliers (standardized residuals, Cook’ distance). IBM SPSS® Statistics for Windows, Version 26 software (IBM Corp., Armonk, NY, U.S.) will be used for all statistical analyses. To correct for multiple testing, Bonferroni-Holm-adjustment of *p*-value will be applied for chi-square tests or independent t-tests. The level of significance for regression analysis will be set at *p* < 0.05 and additionally evaluated based on 95% confidence intervals (CI).

### Dissemination

For the dissemination of the study results scientific publication in national and international journals as well as appropriate conference presentations are planned. With regard to the implementation of the training program after the end of the trial, cooperation will be developed with local sports clubs or biking associations addressing older persons. In addition, the trained instructors could offer the intervention on a private basis.

## Discussion

Cycling as an important component of mobiliy is becoming increasingly popular among older people, especially with regard to e-bike use [13]. As a result of this development, there is a growing need for concepts that promote safer cycling, taking into account age-related functional impairments, and support a reduction in the risk of accidents and the severity of their consequences. Furthermore, as cycling may be a valuable alternative to driving cars with respect to the climate changes, it is of utmost interest to promote safe cycling in all stages of life.

To the best of the authors’ knowledge, there are hardly any evaluated training concepts for increasing the cycling competence of older people. An intervention study by Hagemeister et al. [26] evaluated the effect of a training program on various bicycle-specific abilities, but the training took place without a bicycle and did not specifically include older people with subjective uncertainties or fears when cycling. Based on this research gap, in our study a multi-component intervention was developed that focuses on bicycle-specific skills and the increasing challenge of complex traffic situations in older age [35, 36]. Furthermore, it includes general functional training [37]. In order to meet the needs of older cyclists with uncertainties or re-entrants with a longer cycling break, individual strategies for coping with dangerous situations and fears will be practiced in the training. The holistic intervention approach was developed by an interdisciplinary team of motor scientists, gerontologists and experienced bicycle trainers. By designing the intervention as a course concept, future implementation in existing course structures of sports or cycling clubs would be easily feasible if the intervention proves to be effective.

With a view to promoting and maintaining mobility in older age [38], it would be desirable to provide the intervention to those older people who no longer dare to ride a bicycle due to insecurity, although they would like to do so [39, 40]. Unfortunately, it is not possible, to address these people, because SiFAr’s participants have to be able to get to the training by bicycle on their own for personnel and economic reasons. This might be the main limitation of the study. Furthermore, the missing blinding of the outcome assessors regarding randomization might be a further limitation.

Bicycle type [41] and sex [42] are characteristics that could potentially influence cycling skills. The two characteristics are used as randomization factors in the SiFAr trial to avoid an unequal distribution of characteristics between the IG and the aCG. Thus, a balance of the cofactors should be guaranteed. To the best of the authors’ knowledge, there are hardly any intervention studies to date that have addressed both types of bicycles. This could be due to the fact that the trend towards e-bike use has especially increased in recent years [10–12]. In the study sample of Hagemeister et al. [26], which conducted a training program to promote cycling competence, only two participants were users of motorized bicycles. Although these small number did not allow to analyse differences between users of e-bikes and classic bicycles, the results confirm that the cycle course is feasible with e-bikes. Since a slightly adapted version of this cycle course is used in SiFAr to evaluate the outcome cycling competence, the results of Hagemeister et al. [26] can be considered as a first pilot test regarding e-bikes. From the expectation that a larger number of e-bike users will be recruited for SiFAr, the following arguments can be deduced for randomizing participants according to bicycle type: First, both types of bicycle could place different demands on cycling skills and may therefore systematically influence performance in the cycling course. For example, participants in a study by Haustein et al. [43] on the perceived safety among Danish e-bike riders reported problems in maintaining balance due to the weight of the bike and regulating speed appropriately. It is therefore conceivable that certain tasks in the cycle course, such as slalom and slow driving, may be more challenging for e-bike-riders, while tasks such as the narrow lane may be easier to master due to the stability and speed [44]. Second, studies indicate differences in user motivation between e-bike riders and conventional cyclists [45]. Physically fitter people and frequent cyclists seem to be more likely to ride normal bikes and show less interest in e-bikes, while less active cyclists are more likely to use an e-bike. However, with regard to safer cycling behavior, no differences between e-bike riders and classic cyclists emerged in a study by Langford et al. [46]. Possible differences in the cycling skills of e-bike riders and classical cyclists could be caused less by the type of bicycle or behavioral aspects than by the different user profiles.

To control a possible influence of the bicycle type beyond randomization, we document whether a participant owns more than one bicycle or bicycle type. Participants who own both types of bicycles are asked to choose one of them for the duration of the study. Since studies indicate that it is necessary to familiarize oneself with the e-bike before using it in demanding traffic situations [43, 47, 48], SiFAr is also intended to specifically address beginners with the e-bike.

Due to the physiological differences in strength and endurance performance between women and men [49], sex is a classic factor that is considered in exercise interventions. However, in terms of cycling skills, we believe that potential sex differences are more likely to be expected at the behavioral level. Studies revealed, for example, that sex influences bicycle use. Women in Germany seem to ride their bicycles less often [13], which could be explained by different motivations for bicycle use. Some studies indicate that men seem to use the bicycle both for recreational and for utilitarian purposes, while women use it mainly for recreational reasons [42, 50]. This may be due to traditional role allocations, which make men more likely to be “active road users”, e.g. because of commuting to work [51], especially in the older generations. However, as people reach retirement age, the purpose of bicycle use in old age seems to converge between men and women [13]. Le et al. [42] also showed similarities between men and women in terms of reported barriers to bicycle use, such as avoiding riding on the road without dedicated cycle paths. With regard to self-reported uncertainties when cycling, age in particular could play a role alongside sex [43]. In the cycling monitor Germany [52], a representative online survey, older persons report more often about uncertainties when riding a bicycle compared to younger ones. Despite possible differences in the baseline level of cycling competence, which is taken into consideration through randomization by sex, we therefore assume that the intervention increases the cycling competence of older people regardless of sex.

The target number of cases of SiFAr is sufficient to identify a potential significant improvement in cycling skills through the intervention. Despite this planning, the current situation due to the coronary pandemic is a difficult to predict factor that could affect various components of the study: In the coming year 2021, there may still be contact or exit restrictions that limit or delay recruitment. In addition, there may be restrictions regarding the number of participants in course programs.

The risk of adverse health events (e.g. falls) during the participation in the intervention and assessments of SiFAr is considered low due to trained personnel and safety precautions. Nevertheless, participants are informed prior to inclusion in the study about the possible risk of bicycle falls and related injuries.

In conclusion, this manuscript describes the protocol of the ongoing study SiFAr. The SiFAr trial will investigate the effects of a multi-component exercise intervention on cycling skills in community-dwelling older persons by choosing a holistic approach. The 3 months progressive intervention addresses daily cycling skills as mounting and dismounting of the bike, braking, and turning left. Regarding the physiological and cognitive changes with aging, the training includes strength and balance exercises as well as dual task situations. Psychological aspects of cycling as concerns or fear of falling are also addressed. Longitudinal follow-up will provide additional information on the potential long-term effectiveness of the intervention.

The SiFAr course concept could help increase the safety of older people in road traffic, boost their confidence in their cycling skills and thus reduce the risk of accidents. Furthermore, it could promote the maintenance of mobility and independence into old age. If the effectiveness of the intervention can be demonstrated, the courses could easily be made available to a broad public through the standardized structure and a train-the-trainer approach.

## Supplementary Information

**Additional file 1.** The additional file ‘WHO Trial Registration Data Set’ provides the standardized overview of the trial.

## Data Availability

The datasets used and/or analysed during the current study available from the corresponding author on reasonable request.

## Abbreviations

aCG: Active control group
CI: Confidence intervals
FAU: Friedrich-Alexander-Universität Erlangen-Nürnberg, Germany
FES-I: Falls Efficacy Scale- International Version
HRP: Health related presentations
IBA: Institute of Biomedicine of Aging
IG: Intervention group
MEPC: Multi-component exercise related to cycling
MoCA: Montreal-Cognitive Assessment
SiFAr: Safer cycling in older age
SPPB: Short Physical Performance Battery
TBD: To be done
TBU: To be updated
TMT: Trail Making Test
VAS: Visual Analogue Scale

## Footnotes

1 For this paper the term e-bike will be used exclusively to refer to electrically-assisted bicycles which require the rider to pedal and with pedal assistance up to 25 km/h

2 R Core Team (2020). R: A language and environment for statistical computing. R Foundation for Statistical Computing, Vienna, Austria. URL https://www.R-project.org/.
