# Supplementary material for: Safer cycling in older age (SiFAr): a protocol of a randomized controlled trial
Source: BMC Geriatr. 2021 Oct 12;21:546. doi: 10.1186/s12877-021-02502-5 (PMC8507335; doi:10.1186/s12877-021-02502-5)
Supplement: Supplementary file 1 — Additional file 1. The additional file ‘WHO Trial Registration Data Set’ provides the standardized overview of the trial. [file 12877_2021_2502_MOESM1_ESM.docx]

| **World Health Organization Trial Registration Data Set** |  |
| --- | --- |
| **Item** | **Information** |
| 1. Primary registry and trial-identifying number | ClinicalTrials.gov: NCT04362514 |
| 2. Date of registration in primary registry | 27 April, 2020 |
| 3. Secondary identifying numbers | ethic committee approval number: 22_20B |
| 4. Sources of monetary or material support | ADAC Stiftung |
| 5. Primary sponsor | University of Erlangen-Nürnberg Medical Faculty |
| 6. Secondary sponsor(s) | N.A. |
| 7. Contact for public queries | Dr. Ellen Freiberger, Dr. Robert Kob;  [iba-kontakt@fau.de](mailto:iba-kontakt@fau.de);  +49 911/5302-96150;  Institute for Biomedicine of Aging, Kobergerstr. 60, 90408 Nuremberg, Germany |
| 8. Contact for scientific queries | Dr. Hanna Maria Siebentritt, Veronika Keppner;  [hanna.siebentritt@fau.de](mailto:hanna.siebentritt@fau.de), veronika.keppner@fau.de;  +49 911/5302-96163;  Institute for Biomedicine of Aging Kobergerstr. 60, 90408 Nuremberg, Germany |
| 9. Public title | Safer Cycling in Older Age (SiFAr) |
| 10. Scientific title | Safer Cycling in Older Age (SiFAr) |
| 11. Countries of recruitment | Germany |
| 12. Health condition(s) or problem(s) studied | Older adults, cycling competence, community-dwelling |
| 13. Intervention(s) | Intervention group: MEPC- Multi-component exercise program related to cycling with and without bikes. Training period will last over 3 months with 8 sessions à 60 minutes. Training sessions will address motor competence (balance, strength, cycling skills and techniques) and cognitive skills required during cycling. Furthermore, fall-related psychological concerns will be addressed.  Active Comparator: HRP- 3 Health related presentations with focus on aging and a duration of 60 minutes |
| 14. Key inclusion and exclusion criteria | Age: 65 – 100 years; Sex: All  Inclusion criteria:  living in the area Nürnberg-Fürth-Erlangen, Bavaria, Germany  participant is either 1) a beginner with the e-bike or has 2) self-reported insecurity or is 3) a re-entrant in cycling  Exclusion criteria:  (medical) conditions that prevent regular and safe participation in the intervention |
| 15. Study type | Interventional  Allocation: 1:1 randomized (blocksize 2-4; couples randomized together), stratified for sex and bicycle type (e-bikes/unmotorized bicycle)  Intervention model: parallel assignment  Masking: unblinded  Primary purpose: improvement of cycling competence |
| 16. Date of first enrollment | June 2020 |
| 17. Target sample size | 200 |
| 18. Recruitment status | Recruiting |
| 19. Primary outcome(s) | Number of faults in a standardized cycling course  (Time Frame: changes over a period of 3 months and after a follow-up period of 6-9 and in some cases 18-21 months) |
| 20. Key secondary outcome(s) | N.A. |
